# The Rho/MRTF-pirin axis: a promising target for overcoming melanoma drug resistance

**DOI:** 10.3389/fphar.2025.1571933

**Published:** 2025-07-16

**Authors:** Xiaoyan Chen, Junfeng Zhang, Bao Zhang, Zhi Li

**Affiliations:** ^1^ Department of Rehabilitation, Taihe Hospital, Hubei University of Medicine, Shiyan, Hubei, China; ^2^ Medical College of Nanchang Institute of Technology, Nanchang, Jiangxi, China; ^3^ Putuo Hospital, Shanghai University of Traditional Chinese Medicine, Shanghai, China; ^4^ Hubei Provincial Clinical Research Center for Umbilical Cord Blood Hematopoietic Stem Cells, Taihe Hospital, Hubei University of Medicine, Shiyan, China

**Keywords:** melanoma, drug resistance, Rho/MRTF, apoptosis, pirin

## Background

Melanoma is an aggressive malignant skin neoplasm that is susceptible to distant metastases (Kostaki et al., 2022). This has been a major domain of cancer research for numerous years. The ongoing advancement of targeted therapy and immunotherapy has enhanced the overall survival rate of melanoma patients; yet, drug resistance remains a significant barrier in clinical treatment (Poulikakos et al., 2022; Goswami et al., 2024). Study by Foda et al. (2025) Mechanistic insights into Rho/MRTF inhibition-induced apoptotic events and prevention of drug resistance in melanoma: implications for the involvement of pirin, highlighting the significance of the Rho/MRTF pathway in the development of melanoma drug resistance (Foda et al., 2025). We examine the efficacy of chemical CCG-257081, which obstructs the Rho/MRTF pathway, in preventing and surmounting drug resistance through the induction of apoptosis. This article evaluates the primary findings of the research, including its significance, novelty, and potential avenues for future research.

Melanoma is extremely aggressive and possesses significant metastatic potential, posing a substantial threat to patient survival (Swords et al., 2024). Over the past decade, targeted therapies like BRAF inhibitors (e.g., vemurafenib) and MEK inhibitors have markedly extended the survival of many patients; nonetheless, the majority of patients acquire medication resistance within a few months, resulting in a swift reduction in efficacy (Rubio-Rivas et al., 2020; Guo et al., 2021; Lim et al., 2023). This resistance may arise from a limited quantity of pre-existing resistant cells within the tumor or may develop progressively over the treatment process. Research indicates that around fifty percent of resistant melanoma cells exhibit markedly elevated Rho/MRTF pathway activity (Foda and Neubig, 2023), suggesting that this route is integral to the mechanism of drug resistance. The primary focus of melanoma treatment research is how to effectively inhibit this route to delay or reverse medication resistance. Consequently, Foda et al. examined CCG-257081 as a prospective Rho/MRTF inhibitor.

## Key evidence and findings

The researchers assessed the impact of CCG-257081 on melanoma cells from several angles. The YUMMER_P (vemurafenib-sensitive) and YUMMER_R (vemurafenib-resistant) mouse melanoma cell lines were subjected to treatment with vemurafenib, CCG-257081, or a combination of both across various experimental groups. Cell fusion and caspase-3/7 activation were assessed using real-time cellular imaging. To ascertain the dynamic alterations in cellular apoptosis. The extent of PARP cleavage was assessed via Western blotting to further validate the incidence of apoptosis. The *in vivo* anticancer efficacy of CCG-257081 was assessed in a murine model, employing immunohistochemistry labeling of tumor tissue to examine alterations in critical markers, including Ki-67 and cleaved caspase-3. The researchers concentrated on the distinct enantiomers of the chemical (R and S) and evaluated their capacity to bind pirin and modulate ACTA2 gene expression by fluorescence polarization competition assays and gene expression assays. The variety of experimental designs enhances the credibility of the results and offers numerous corroborative avenues for investigating the specific mechanism. The authors indicate in the commentary that the absence of PARP cleavage with Vem combined with CCG-257081 necessitates additional investigation. Is it possible that the rate of apoptosis during combination therapy is excessively rapid, preventing the timely accumulation of the PARP cleavage product?

CCG-257081, either independently or in conjunction with vemurafenib, markedly suppressed the proliferation and triggered apoptosis of YUMMER_P and YUMMER_R cells at the cellular level. The combination of vemurafenib and CCG-257081 markedly enhanced cell apoptosis. CCG-257081 decreased Ki-67 expression and increased cleaved caspase-3 staining in the murine model, hence corroborating its *in vivo* anti-tumor efficacy.

The two enantiomers of CCG-257081 exhibit differing activity. The S-enantiomer exhibits a stronger affinity for pirin and demonstrates more potency in suppressing TGFβ-induced ACTA2 gene expression. The enantiomeric distinction offers guidance for the subsequent optimization of chemical structure and enhancement of activity. Recent structural and biophysical investigations elucidate the reasons for the superior affinity of the S-enantiomer of CCG-257081 for pirin. Molecular docking and binding tests demonstrate that the S-enantiomer achieves a superior fit within the pirin active site, forming critical hydrogen bonds and hydrophobic interactions that stabilize the inhibitor-protein complex (Lisabeth et al., 2019). The S-enantiomer’s orientation facilitates interaction with essential amino acid residues in the binding pocket, hence augmenting its inhibitory effectiveness relative to the R-enantiomer. The data indicate that stereochemistry is crucial in ligand binding and selectivity. This structural understanding can inform the rational design of future Rho/MRTF pathway inhibitors by optimizing enantiomeric configuration and strengthening interactions with pirin, resulting in molecules with superior efficacy, selectivity, and safety for melanoma and other malignancies.

Pirin plays a significant influence in melanoma’s responsiveness to MRTF inhibitors. The downregulation of pirin enhances the inhibitory effect of MRTF signaling, resulting in reduced proliferation and survival of melanoma cells^10^. This indicates that pirin may function as a unique adjuvant treatment target, offering an improved technique for augmenting Rho/MRTF signaling suppression. Recent mechanistic investigations have clarified that pirin acts as a transcriptional co-regulator in melanoma cells, directly engaging with MRTF-A (myocardin-related transcription factor A). Pirin can affect the subcellular location of MRTF-A, enhancing its nuclear retention and consequently regulating the transcription of Rho/MRTF downstream target genes associated with cytoskeletal remodelling, cell motility, and cell survival (Lisabeth et al., 2019). This interaction is believed to refine the transcriptional output of the Rho/MRTF pathway, enhancing cellular plasticity and resistance to therapy. Moreover, recent evidence suggests that the pirin-MRTF axis may be influenced by upstream oncogenic signalling events, particularly those associated with BRAF or NRAS mutations, prevalent in melanoma. Initial studies indicate that pirin expression and its regulatory role on MRTF are significantly elevated in BRAF-mutant melanoma cells, potentially making these subtypes more vulnerable to concurrent targeting of Rho/MRTF and pirin (Ibrahim et al., 2025). The precise function of pirin in resistance mechanisms across various genetic backgrounds (e.g., BRAF versus NRAS mutations) has yet to be thoroughly elucidated, necessitating additional research to clarify these subtype-specific effects.

## Implications for adjuvant therapy decisions

The research conducted by Foda et al. thoroughly investigates the Rho/MRTF pathway’s involvement in melanoma treatment resistance and suggests the potential of CCG-257081 as an inhibitor of this system to postpone or reverse medication resistance. This study elucidates the disparities in the activities of CCG-257081 enantiomers, establishing a foundation for molecular modification and enhancement of anticancer efficacy. The notion of pirin as a prospective adjuvant treatment target enhances the comprehension of melanoma drug resistance mechanisms and introduces novel concepts for integrated multi-target interventions. These discoveries hold significant significance for the advancement of more efficient and focused therapy protocols.

## Future research directions

### Key questions arising directly from the Foda et al. Study

The Foda et al. study identifies CCG-257081 as a promising agent for overcoming targeted therapy resistance in melanoma, raising several critical questions that warrant immediate investigation. While Pirin was identified as the direct target, its precise mechanism in melanoma cells needs deeper elucidation, particularly regarding its role in proliferation and apoptosis. A key next step is to design rational combination regimens with existing kinase inhibitors, building on the study’s finding that CCG-257081 can resensitize cells to BRAF/MEK inhibitors. This requires investigating the molecular basis for synergy and defining optimal dosing and sequencing to maximize efficacy (Kang et al., 2025). To better predict clinical outcomes and pave the way for clinical trials, it is essential to validate these findings in more advanced preclinical models, including diverse cell lines, varied animal models, and patient-derived organoids, which more faithfully replicate the clinical setting. The superior activity of the S-enantiomer also suggests that further chemical modifications could yield even more potent compounds.

### Broader research directions informed by other recent findings in the field

Integrating the Foda et al. findings with broader knowledge from the field opens up new, exploratory research avenues. For instance, recent studies on related CCG compounds suggest they can induce apoptosis through alternative, Rho/MRTF-independent mechanisms, such as directly modulating mitochondrial function and inducing reactive oxygen species (ROS) (Lisabeth et al., 2019). It is crucial to explore whether CCG-257081 shares these off-target effects, which could provide additional strategies to overcome resistance. Furthermore, the importance of the Rho/MRTF pathway extends beyond melanoma to other solid tumors like pancreatic and breast cancer, where it drives metastasis. Since CCG-257081 has shown preclinical efficacy in these models, strategies developed for melanoma may be transferable to other aggressive cancers (Beljkas et al., 2024; Sun et al., 2025). Finally, a major unexplored opportunity lies in investigating the synergy between CCG-257081 and immunotherapy. As resistance to immune checkpoint inhibitors is a significant clinical hurdle, determining whether MRTF inhibition can modulate the tumor immune microenvironment and enhance immunotherapy efficacy is a critical future direction that could benefit a broad patient population.

## Conclusion

This investigation of CCG-257081 offers novel insights into the issue of melanoma treatment resistance. Inhibiting Rho/MRTF signaling by pharmacological agents might markedly cause apoptosis in tumor cells, hence potentially augmenting current targeted or immunotherapy approaches. The putative auxiliary role of pirin in this process also suggests a pathway for multi-target therapeutic techniques. Despite remaining unsolved concerns, these findings represent a significant advancement in understanding drug resistance mechanisms and melanoma treatment, offering a realistic research foundation and ample opportunity for creativity in enhancing current therapies and generating novel pharmaceuticals.
